# Systemic distribution of different low pathogenic avian influenza (LPAI) viruses in chicken

**DOI:** 10.1186/1743-422X-10-23

**Published:** 2013-01-17

**Authors:** Jacob Post, Eveline D de Geus, Lonneke Vervelde, Jan BWJ Cornelissen, Johanna MJ Rebel

**Affiliations:** 1 Central Veterinary Institute of Wageningen UR, P.O. Box 65, Lelystad,, 8219 PH,, The Netherlands; 2 Faculty Veterinary Medicine, Dept. Infectious Diseases and Immunology, Utrecht University, Yalelaan 1, Utrecht, 3584 CL, The Netherlands

**Keywords:** Low pathogenic avian Influenza, Chickens, Systemic distribution

## Abstract

**Background:**

Since we were able to isolate viable virus from brain and lung of H7N1 low pathogenic avian influenza virus (LPAIV) infected chickens, we here examined the distribution of different LPAIV strains in chickens by measuring the viral AI RNA load in multiple organs. Subtypes of H5 (H5N1, H5N2), H7 (H7N1, H7N7) and H9 (H9N2), of chicken (H5N2, H7N1, H7N7, H9N2), or mallard (H5N1) origin were tested. The actual presence of viable virus was evaluated with virus isolation in organs of H7N7 inoculated chickens.

**Findings:**

Viral RNA was found by PCR in lung, brain, intestine, peripheral blood mononuclear cells, heart, liver, kidney and spleen from chickens infected with chicken isolated LPAIV H5N2, H7N1, H7N7 or H9N2. H7N7 virus could be isolated from lung, ileum, heart, liver, kidney and spleen, but not from brain, which was in agreement with the data from the PCR. Infection with mallard isolated H5N1 LPAIV resulted in viral RNA detection in lung and peripheral blood mononuclear cells only.

**Conclusion:**

We speculate that chicken isolated LPAI viruses are spreading systemically in chicken, independently of the strain.

## Findings

Avian influenza (AI) A is a highly heterogeneous group of viruses with varying pathogenicity in different species. Influenza virus subtypes have gained sufficient adaptive molecular changes to become established in domestic poultry and cause mild to severe disease [1-3]. AI is classified by the world organization for animal health into two pathotypes, low pathogenicity avian influenza viruses (LPAIV) and high pathogenicity avian influenza viruses (HPAIV), based on their virulence in chickens. Pandemic influenza outbreaks of HPAIV in poultry pose a significant threat to public health as highlighted by the emergence of H5N1 HPAIV [4]. In general, the HPAIV emerged from a H5 or H7 LPAIV subtype that was circulating in chickens or turkeys [1]. LPAIV haemagglutinin (HA) lacks the polybasic cleavage site that characterizes most HPAIV. The monobasic cleavage site of LPAIV favors trypsin-like proteases, which are thought to be secreted only by cells of the respiratory and intestinal tract. However, some chicken-isolated LPAIV strains have been isolated from a limited number of other tissues including the pancreas, kidneys and oviduct of intranasally (i.n.) or intratracheally (i.t.) inoculated chickens [5]. In addition, we were able to isolate viable virus from brain of H7N1 LPAIV inoculated chickens [6]. With the introduction of PCR several reports indicate systemic distribution of mRNA of LPAIV strains like H7N1 and H9N2 [6-8]. In order to investigate whether systemic distribution of LPAIV was restricted to some individually reported strains, we tested the systemic distribution of four LPAIV chicken (C-LPAIV) and one mallard (M-LPAIV) isolate, using PCR in chicken.

H7N1 LPAIV (A/Chicken/Italy/1067/99) was a gift from Dr. Ilaria Capua (Istituto Zooprofilattico Sperimentaledelle Venezie, Italy). H5N1 LPAIV (A/Mallard/Italy/3401/05), H5N2 LPAIV (A/Chicken/Pennsylvania/21525/83) and H7N7 LPAIV (A/Chicken/Netherlands/06022003/06) were kindly provided by Dr. Guus Koch (Department of Virology, Central Veterinary Institute of Wageningen UR, The Netherlands). H9N2 (A/Chicken/Saudi Arabia/SP02525/3AAV/2000) was obtained from the Animal Health Service (Deventer, The Netherlands). The viruses were propagated and titrated in the allantoic cavities of 10-day-old SPF chicken eggs to prepare stock virus. For animal experiments, the viruses were diluted in sterile PBS to 10^6^ EID_50_ per ml immediately prior to use. Non-vaccinated AI-free chickens were randomly divided over four (experiment 1) or 2 (experiment 2) groups and housed in floor cages for 3 weeks prior to inoculation. Food and water were provided ad libitum. In compliance with Dutch law, all experiments were approved by the Animal Experimental committee of the institutions, in accordance with the Dutch regulations on experimental animals. For the first experiment one-day-old Lohmann Brown layer chickens were obtained from a commercial breeder (Pronk’s Broederij, Meppel, The Netherlands). Chickens were inoculated with 0.2 ml (2×10^5^ EID_50_) of the LPAIV strains H7N1, H7N7 or H5N1 equally divided between the i.n. and i.t. route. Control chickens were inoculated with 0.2 ml PBS. Six chickens from each group were sacrificed at 2 and 4 days post infection (d.p.i.). The body weight of the chickens was established daily and from all sacrificed chickens gross pathology of the organs was studied. Lung, brain, ileum, blood, liver, kidney, heart and spleen were collected for RNA extraction, snap-frozen in liquid nitrogen and stored at −80°C until use. A piece of about 0.5×0.5 cm tissue was used which correspond to approximately 200 mg. peripheral blood mononuclear cells (PBMC) were isolated from blood by ficol-paque (Amersham Biosciences, Uppsala, Sweden). For the second experiment the chickens were obtained from a commercial breeder and inoculated as in exp. 1 with LPAIV H5N2 or H9N2. Five chickens from each group were sacrificed at 2 and 4 d.p.i. The body weight of the H5N2 LPAIV inoculated chickens was established at 0, 1, 2 and 4 d.p.i.

For RNA isolation, organs were stored in trizol (Invitrogen, Breda, The Netherlands) at −80°C until use. Both methods of preservation were tested and give equal RNA values. RNA isolation of the samples of both experiments was performed using the phenol/chloroform method as previously described [6]. H7N1, H7N7, H5N1 and H5N2 were analyzed using the quantitative PCR (qPCR) [6]. For H9N2 cDNA was generated from 500 ng RNA with reverse transcription using an iScript cDNA Synthesis kit (Biorad, Veenendaal, The Netherlands). H9N2 viral cDNA was amplified with specific primers and conditions as described [6]. RNA samples of day 2 p.i. of H9N2 infected chickens were tested using an one and two step PCR with the primers described. Comparable Ct values for AI load were obtained. Therefore both PCR methods can be used next to each other. As a control, tissue samples of the uninfected chickens were included in each run of the qPCR. Standardization of the PCR was done by equalizing the amount of input RNA with 200 ng. The quality and integrity of the RNA samples was analyzed using the Agilent Bioanalyzer (lab on chip, Agilent). The data were expressed as Ct-45 values, which means that increased values in the figures indicate increased amount of viral RNA and when Ct-45 is equal to zero no viral RNA was detected. Because no Ct values were found for any organs of control chickens tested during a 45 cycles run in these particular PCRs, we considered Ct values ≤ 45 as positive [6]. H7N7 inoculated chickens from the first experiment were used as an example to confirm the presence of progeny virus in tissues where viral RNA was detected by PCR [6]. Briefly, the supernatant of homogenized and clarified organs of six H7N7 inoculated chickens (4 d.p.i.) was divided over five embryonated eggs. The eggs were screened daily and incubated for 7 days. After 7 days or earlier after embryo mortality the allantoic fluid was harvested. The presence of H7N7 virus in the allantoic fluid was tested with the HA-test and sequencing. Differences between the Ct values of the strains were analyzed for statistical significance by the Mann–Whitney U test. For this purpose the Ct value of samples with no detectable virus was set at 45. Differences in weight was analyzed for statistical significance by the Student’s T-test. *P* ≤ 0.05 was considered significantly different.

**Figure 1:**
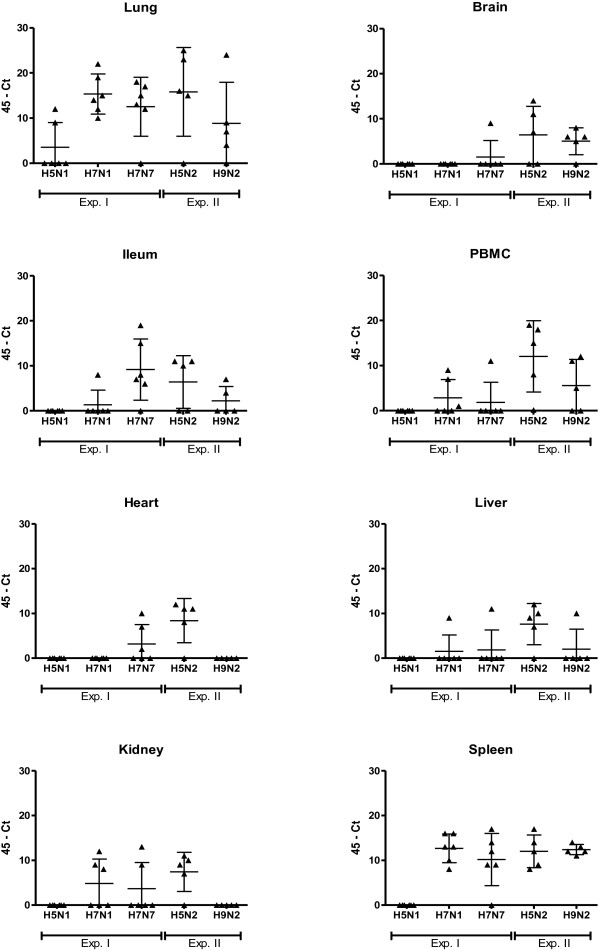
**Scatter plot of 45-Ct values of viral RNA from organs of individual birds at 2 days post inoculation (d.p.i.). **Chickens were inoculated with different LPAIV strains and the presence of viral RNA in organs was examined at 2 d.p.i. by qPCR. The horizontal line represents the mean of 5 or 6 individual birds with SD. Triangles represent the 45-Ct value of individual birds. The data were expressed as Ct-45 values, which means that increased values in the figures indicate increased amount of viral RNA and when Ct-45 is equal to zero no viral RNA was detected. No viral RNA was detected in controls (Ct = 45). M-LPAIV H5N1, C-LPAIV H7N1 and H7N7: n=6. C-LPAIV H5N2 and H9N2: n=5.

**Figure 2:**
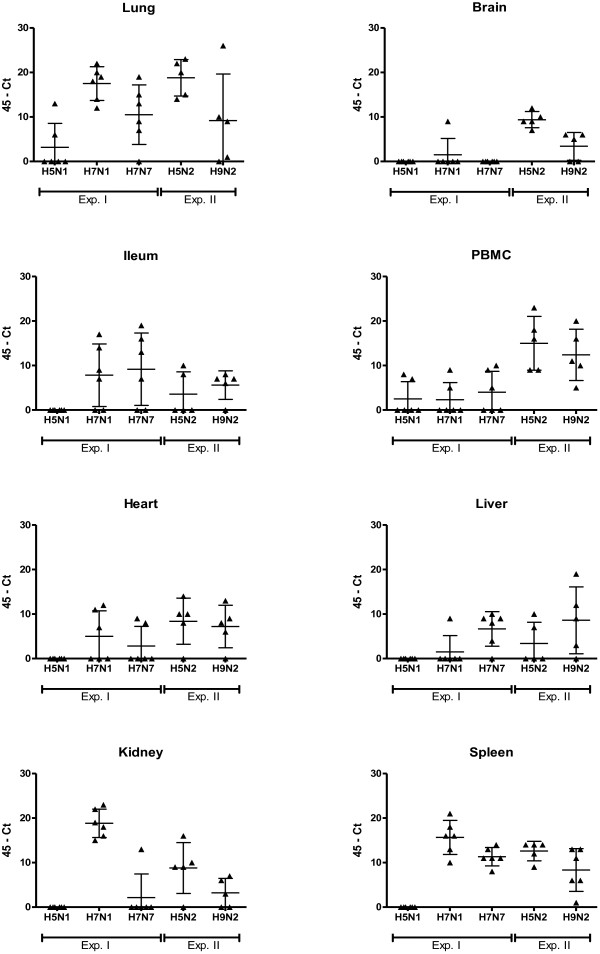
**Scatter plot of 45-Ct values of viral RNA from organs of individual birds at 4 days post inoculation (d.p.i.). **Chickens were inoculated with different LPAIV strains and the presence of viral RNA in organs was examined at 4 d.p.i. by qPCR. The horizontal line represents the mean of 5 or 6 individual birds with SD. Triangles represent the 45-Ct value of individual birds. The data were expressed as Ct-45 values, which means that increased values in the figures indicate increased amount of viral RNA and when Ct-45 is equal to zero no viral RNA was detected. No viral RNA was detected in controls (Ct = 45). M-LPAIV H5N1, C-LPAIV H7N1 and H7N7: n=6. C-LPAIV H5N2 and H9N2: n=5.

To determine whether the systemic detection of LPAIV RNA was restricted to a limited set of strains, we measured systemic distribution of viral RNA from five LPAIV strains using PCR. Subtypes of H5 (H5N1, H5N2), H7 (H7N1, H7N7) and H9 (H9N2), of chicken (H5N2, H7N1, H7N7, H9N2), or mallard (H5N1) origin were tested. Systemic distribution of virus was confirmed by virus isolation of H7N7 C-LPAIV virus particles from organs that were positive for viral RNA by PCR. Low pathogenicity was confirmed by sequencing over the HA cleavage site [6]. Body weight of the chickens was measured as an indicator of severity of the disease after inoculation. Systemic distribution of LPAIV RNA was found for the C-LPAIV strains H7N1, H7N7, H5N2 and H9N2. For these strains viral RNA was found in multiple organs (Figures 1 and 2). In contrast to the C-LPAIV strains, viral RNA of the mallard isolated H5N1 LPAIV strain was only found in the lung at 2 and 4 d.p.i. and in PBMC at 4 d.p.i. Furthermore, the viral RNA load in in the lung and PBMC of H5N1 M-LPAIV inoculated chickens was generally low. In the lung, significant differences were found at 2 d.p.i. (compared to H7N1, H7N7 and H5N2 (*p* ≤ 0.05)) and at 4 d.p.i. (compared to H7N1 and H5N2 (*p* ≤ 0.01)). For PBMC significant differences were found at 4 d.p.i. (compared to H5N2 (*p* ≤ 0.01) and H9N2 (*p* ≤ 0.05)). A clear contrast between M-LPAIV and C-LPAIV was also seen in the viral RNA load in the spleen. While in the majority of the C-LPAIV inoculated chickens viral RNA was detected in the spleen, viral RNA could not be detected in the spleen of H5N1 M-LPAIV inoculated chickens (Figures 1 and 2).

Although viral RNA was detected in most lungs and spleens after inoculation with C-LPAIV strains, differences were found between the C-LPAIV strains. After a H5N2 C-LPAIV infection all chickens become positive for viral RNA and almost all evaluated organs of these chickens were infected (Figures 1 and 2). At 2 d.p.i. a significant higher viral load was found in PBMC (compared to H7N1, H7N7 (*p* ≤ 0.01) and H9N2 (*p* ≤ 0.05)), heart (compared to H7N1 and H9N2 (*p* ≤ 0.01)) and kidney (compared to H9N2 (*p* ≤ 0.01)). At 4 d.p.i. a significant higher viral load was found in brain (compared to H7N1, H7N7 (*p* ≤ 0.01) and H9N2 (*p* ≤ 0.05)) and PBMC (compared to H7N1 (*p* ≤ 0.01) and H7N7 (*p* ≤ 0.05)). A typically high viral RNA load was found in the kidney of H7N1 inoculated chickens at 4 d.p.i. (significant differences compared to H7N7, H9N2 (*p* ≤ 0.01) and H5N2 (*p* ≤ 0.05)), in the ileum of H7N7 inoculated chickens at 2 d.p.i. (significant differences compared to H7N1 (*p* ≤ 0.05)) and in the PBMC of H9N2 inoculated chickens (significant differences compared to H7N1 and H7N7 (*p* ≤ 0.05)). Figures 1 and 2 show that not all chickens were positive in brain (H7N1 and H7N7), heart (H7N1 and H9N2) and kidney (H9N2). The cause of the differences in distribution between the C-LPAIV strains has not been studied here, but might for instance be related to receptor affinity [9,10] or replication efficiency as suggested for differences between HPAIV [11].

**Figure 3:**
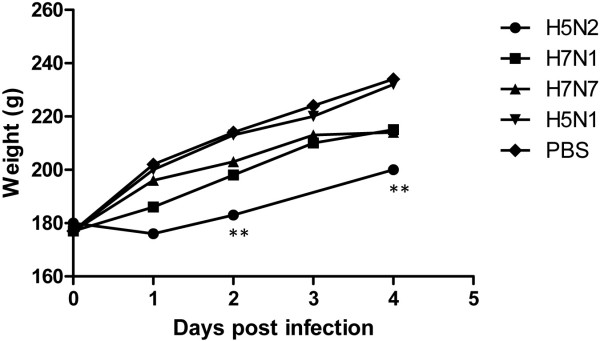
**Weight gain during the experimental period. **The bodyweight of the chickens was established pre- and post-infection. Symbols represent the mean of individual chickens. PBS control, M-LPAIV H5N1, C-LPAIV H7N1 and H7N7: n=6. C-LPAIV H5N2 and H9N2: n=5. **: P ≤ 0.01.

In agreement with the viral RNA distribution, a delay in bodyweight gain was found for C-LPAIV H5N2, H7N1 and H7N7 but not for H5N1 M-LPAIV infected chickens (Figure 3). The decrease in weight gain was only significant for H5N2 C-LPAIV, which corresponded with the highest number of organs in which viral RNA was detected. Although bodyweight was not measured after H9N2 inoculation in this experiment, weight loss was previously found after infection with this virus strain (pers. comm. C.A. Jansen) and also in relation to other H9N2 strains [12-14].

In contrast to the review of Spickler et al. (2008) we here show a wide viral RNA distribution over multiple organs of different LPAIV strains isolated form chickens. The differences in amount of viral RNA between strains in tissues might indicate that not all C-LPAIV could be detected systemically. However, since our study shows that different LPAI strains were able to spread systemically beyond the respiratory and gastrointestinal tract, systemic distribution of LPAI viruses could very well be a general phenomenon. In a parallel experiment we were able to isolate H7N1 LPAI virus from the chicken brain, where the necessary proteases to replicate are absent [6]. Here we isolated viable H7N7 LPAIV from lung, intestine, heart, liver, kidney and spleen (Table 1). Virus could only be isolated from organs with a high viral RNA load (Ct ≤ 35) as measured with the qPCR. Differences in virus detection between virus isolation and qPCR is considered to be due to sensitivity differences in assays used [6,15]. No virus could be isolated from the brain of H7N7 LPAIV inoculated chickens, which was in agreement with the negative result in the PCR at day 4. Altogether, viral RNA in different organs might implicate the presence of propagated virus.

**Table 1:** Detection of virus in organs of H7N7 LPAIV inoculated chickens by virus isolation and HA

**Organ**	**Virus present (pos. chickens/total tested)**
**Lung**	++ (4/6)
**Brain**	**-** (0/6)
**Ileum**	**++** (3/6)
**PBMC**	N.D.
**Heart**	**+** (2/6)
**Liver**	**+** (4/6)
**Kidney**	**+** (1/6)
**Spleen**	**+** (5/6)

++ virus detected (HA titer of positive samples 128 – 512).

+ virus detected (HA titer of positive samples 16 – 64).

- No virus detected; N.D. not done.

While there is sufficient literature on C-LPAIV, reports studying M- LPAIV infections are scarce. Ladman et al. [16] examined the upper respiratory and the intestinal tract of turkeys and chickens after inoculation of different LPAI viruses, including the mallard isolates H5N1 and H7N3. H5N1 LPAIV could only be isolated from the upper respiratory tract of turkeys, while H7N3 LPAIV was isolated from the upper respiratory and the intestinal tract of both turkeys and chickens. The fact that H5N1 LPAIV was detected in turkeys and not in chicken might be explained by a difference in susceptibility for AI between turkeys and chickens [17]. Although the tissue tropism of the mallard isolates LPAIV H5N1 and H7N3 might differ in chicken, we speculate that differences between H5N1 M- LPAIV and the C- LPAIV subtypes that were tested in our study were related to differences in adaptation to the host. Starting from the primary infected organs, systemic distribution is among others facilitated by lymphocytes [18,19]. In chickens with viral RNA of H5N1 M-LPAIV in the lung (Figure 2) we also found viral RNA in PBMC at 4 d.p.i., confirming the relation between the level of viral RNA in the primary infected lung and possibilities for systemic distribution via blood cells. In the primary infected lung, where the necessary proteases for LPAIV replication are available, H5N1 M-LPAIV RNA could not be measured in 4 out of 6 chickens on day 2 and 4 (Figures 1 and 2). When the experiment was repeated the results were largely identical (data not shown) and the absence of viral RNA in the lung of H5N1 M-LPAIV infected chickens was therefore probably not related to the experimental infection method. The absence of virus in the lung (and other organs) of H5N1 M-LPAIV infected chickens might be related to impaired replication opportunities of the virus in the non-mallard host. Why some H5N1 M-LPAIV infected chickens did have a high RNA load in the lung is unclear.

In summary, RNA of a panel of LPAI viruses isolated from chickens can be detected in multiple organs of chickens. Viral RNA might indicate viable virus in organs beyond the respiratory tract as was shown for H7N1 and H7N7 LPAIV. Differences in systemic distribution between the LPAIV strains isolated from chickens exist but larger differences are found between the chicken isolated strains and the mallard isolated strain. These differences are likely to be dependent on host preference and might eventually be related to replication efficiency of the virus in different hosts.

## Abbreviations

LPAIV: Low pathogenic avian influenza virus; C-LPAIV: Chicken isolated low pathogenic avian influenza virus strain; M-LPAIV: Mallard isolated low pathogenic avian influenza virus strain; d.p.i: Days post infection; i.n: Intranasal inoculation; i.t: Intratracheal inoculation.

## Competing interests

The authors declare that they have no competing interests.

## Authors’ contributions

EDdG carried out animal experiment II and participated in the interpretation of the data. LV was responsible for the design of animal experiment II. JBWJC assisted in animal experiment I. JMJR was responsible for the study design and interpretation of the data. JP was responsible for animal experiment I, carried out the qPCRs, participated in the interpretation of the data and drafted the manuscript. All authors read and approved the final manuscript.
